# Probing both sides of the story

**DOI:** 10.1073/pnas.2212419119

**Published:** 2022-09-07

**Authors:** Christopher M. Yip

**Affiliations:** ^a^Department of Chemical Engineering and Applied Chemistry, University of Toronto, Toronto, ON M5S 3E9, Canada;; ^b^Institute of Biomedical Engineering, University of Toronto, Toronto, ON M5S 3E9, Canada;; ^c^Department of Biochemistry, University of Toronto, Toronto, ON M5S 3E9, Canada;; ^d^Donnelly Centre for Cellular and Biomolecular Research, University of Toronto, Toronto, ON M5S 3E9, Canada

As the saying goes—there are always two sides to a story. This is never truer than in the context of imaging, where our ability to directly visualize and quantify how molecules interact to form functional structures has long been a key goal in fields ranging from materials science to biology, physics, and chemistry. Such insights, including whether assembly drives conformational changes or vice versa, have tremendous potential in the realm of structural biology because of their relevance, in particular, to diseases associated with protein aggregation, misfolding, or fibril formation. Our ability to ask and, hopefully, answer these and related questions has been certainly aided by developments in single-molecule imaging and characterization tools. While approaches such as localization and superresolution microscopies have garnered significant attention of late, a key challenge is that they are based on localizing a particular signal. In the case of fluorescence techniques, interpretation of that localization then becomes dependent on the characteristics of the fluorophore. What if one could combine multiple complementary approaches together that can then provide a more comprehensive perspective on these stories? In PNAS, Zhao et al. (1) describe one such strategy using nano-Fourier transform infrared spectroscopy (nano-FTIR).

While techniques such as cryoelectron microscopy can provide exquisite three-dimensional subnanometer insights, they are not well suited for following dynamic changes in conformation, shape, and structure in real time. Moreover, they are, effectively, ensemble approaches that rely on reconstruction of many individual images. Scanning probe microscopy (SPM) has been perhaps the key tool for mapping molecular structures and assemblies and, in particular, following dynamic changes, largely because of its ability to operate under a wide range of imaging conditions, including in fluid. Indeed, recent work has demonstrated how advances in high-speed atomic force microscopy (AFM) have now enabled direct imaging of conformational changes in septins and membrane proteins (2, 3). Although impressive, the contrast in an AFM image is based on relative changes in shape, which means that confirming the conformational state or structure of a protein or assembly means either knowing it a priori or being able to trigger a change in situ and inferring what has happened, often based on a separate series of experiments. Developing a platform and/or technique that allows one to provide independent confirmation on the nanoscale or single-molecule scale of a given individual molecule’s chemistry, conformational state, or secondary structure opens up tremendous opportunities, especially in the fields of structural biology, materials science, and chemistry. The coupling of single-molecule fluorescence with SPM, for example, represents one such approach, with recent work clearly demonstrating the opportunities and potential of such an integration and how the field has grown (4–6). Albeit powerful, key challenges with this approach are the requirement for intrinsic fluorescence or addition of an extrinsic fluorophore, and that insights into molecular conformation and structure are largely restricted to regions near the fluorophore itself. One compelling alternative strategy would be to explore opportunities in labelless approaches, such as vibrational spectroscopy (infrared, Raman). These platforms can provide exquisite insights into molecular conformation and structure and, when appropriate, dynamics; however, these are often ensemble measurements with spectral imaging typically diffraction limited, at least as conventionally practiced. With the advent of SPM, however, researchers are presented with an intriguing opportunity to explore subdiffraction limit (or superresolved) vibrational spectroscopy using the AFM tip either as a scattering source (scanning scattering near-field optical microscope [s-SNOM], tip-enhanced Raman [TERS]) or to monitor localized photothermal (PT) expansion–PT-induced resonance (7, 8). These approaches are providing researchers with compelling new perspectives on protein aggregation, fibril formation, polymer films, and lipid membrane domain structures (8–13). However, a key challenge remains in nano-IR spectroscopy—namely, the ability to perform these measurements in liquid. Zhao et al. (1) describe a creative way of addressing these concerns in an s-SNOM platform, or what they describe as nano-FTIR, showcasing how this can be used to track the assembly and conformational dynamics of S-layer proteins (1).

**Fig. 1.**
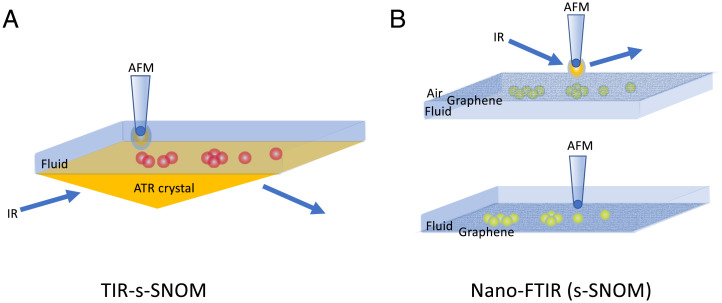
Schematic representation of two tip-enhanced nanospectroscopy platforms. (*A*) TIR-s-SNOM with the AFM tip positioned to scan the surface of an ATR prism, enabling simultaneous image and spectral acquisition; (*B*) nano-FTIR/s-SNOM configuration using a graphene membrane as a substrate, with the FTIR and AFM datasets collected separately.

In the past, a number of groups have sought to couple IR and AFM by creating a hybrid platform that enables attenuated total reflectance (ATR) IR spectroscopy in fluid while using the AFM to follow the dynamics and structures present on the surface of the ATR crystal (14, 15) While this approach leverages the AFM’s ability to operate in fluid with the relative ease of operating a conventional fluid ATR accessory, there remain some key caveats. These include the spectral range associated with the ATR crystal (Ge, Si, ZnSe, diamond), and even how the crystal itself—chemistry, structure, and surface roughness—may affect the sample, especially if one is studying aggregation or binding behavior. Imaging the surface of the ATR crystal, especially during acquisition of spectra, can, in fact, be very informative in terms of explaining time-dependent spectral phenomena. Moreover, in these earlier designs, these platforms do not provide direct correlation of spectral features with specific structures resolved by the AFM tip itself, since the ATR sampling is over a much larger area than what is being imaged by the AFM tip. Regardless, they did demonstrate the potential that exists in such a functional coupling, and specifically illustrated the need to develop approaches that could leverage the high spatial and spectral resolution afforded tip-based nanospectroscopy with the ability to perform these experiments in fluid environments. Interestingly, recent efforts have made headway in this very regard by coupling an s-SNOM configuration using total internal reflection excitation through a ZnSe prism (TIR-s-SNOM), rather than scattering from the tip itself (16). In this inverted approach, it is now simpler to exploit the SPM tip interaction with the evanescent IR field generated at the surface of the ZnSe prism. In this way, one benefits from localization of the near-field effect by the tip but in a manner that removes the challenge of the direct scattering approach described earlier. This is an exciting development that nicely illustrates the evolution of such an integrated approach and offers up a number of important opportunities for studies into biomolecular phenomena and behaviors, including protein dynamics and structures (Fig. 1).

Now, an interesting complement to the TIR-s-SNOM approach is that reported in PNAS by Zhao et al. (1), and previously by Khatib et al. (17), that builds off the concept of creating a sealed sample chamber that is transparent to IR radiation and which enables s-SNOM measurements (nano-FTIR) (18). In this approach, the chamber containing the sample and fluid media is sealed from air by a graphene membrane, with the SPM tip scanning the graphene membrane from the air side. In this configuration, unlike the other tip scattering approaches, the tip does not interact directly with the sample. Rather, in an ingenious strategy, as the tip scans the back side of the membrane, the near-field IR radiation scattered by the tip passes through the graphene membrane to interact with the sample. This strategy nicely addresses the ever-present challenge with any tip-based approach, which is that of sample contamination and/or damage. Furthermore, it, interestingly, may provide a way of answering the issue raised earlier of how the substrate affects the sample itself since graphene itself is atomically smooth with a known structure or two-dimensional pattern, unlike ATR crystals, which are far from smooth on a molecular length scale and which can be damaged during cleaning. In the work by Zhao et al., tracking the adsorption of S-layer proteins to the graphene surface over time revealed subtle changes in the amide I and II band intensities that were attributed to reorientation over time, self-association dynamics, and domain formation. They also reported counter ion-dependent spectral shifts, which they suggested could have been indicative of enhanced H-bonding effects. Unlike the TIR-SNOM work described earlier wherein the topography and the spectral data are acquired simultaneously, in this work, the AFM topography data were acquired in separate experiments, with correlation between the AFM topography and spectral data being inferred, both from the extent of coverage of the graphene surface by the S-layer protein domains and from the size of individual domains. What is perhaps unclear from these studies, however, is the role that the graphene plays in orienting the adsorbing species or influencing their assembly (19). While this may be less of a concern for the proteins of interest in this work, it is certainly well known that ordered substrates, like graphite, can, in fact, facilitate epitaxial growth of peptide fibrils (20–22). Another consideration, especially in the context of membrane proteins, would be how well a membrane-mimetic surface can be created on graphene, although efforts in graphene-based biosensors are certainly encouraging in this regard (23–25).

As with all coupled approaches where the focus is on acquiring multimodal images and spectra, there will always be compromises. For instance, in all the coupled AFM-IR approaches, one consideration that is often not explicitly discussed is the relevant time frame, in the context of both the actual physical phenomena of interest and the accessible instrument time scales. With any tip scattering approach, whether TERS, TIR-SNOM, s-SNOM, or nano-FTIR, one is inherently limited by the tip dwell time required to acquire sufficient spectral signal. This means that the time required to acquire a complete dataset (spectra + AFM image) may be substantial and could, in fact, require rescanning the sample to independently acquire the AFM dataset. These considerations are important when considering the specific experimental system and context. For example, as is seen in the work by Zhao et al. (1), the time scale of S-layer protein assembly and conformational changes was such that the nano-FTIR spectral imaging was able to resolve these dynamic changes. In the coupled non-tip-enhanced ATR-AFM approaches where the instruments are integrated but operating independently, this is no longer an issue. Instead, one is now faced with reconciling how the IR spectra are being acquired for a region much larger than that scanned by the AFM.

Despite these caveats, it is quite clear that such innovative strategies, which enhance the spatial resolution of vibrational spectroscopies to well below the classic diffraction limit, are providing researchers with unique glimpses into conformational dynamics at the level of individual molecular assemblies in near real time and under nominally real-world conditions. This opens up a whole new world of possibilities for studies into more complex phenomena, including membrane dynamics, protein–membrane interactions, and perhaps even structures in live cells. For example, it would be intriguing to explore the potential of TIR-SNOM and nano-FTIR/s-SNOM approaches for tackling membrane-active or membranolytic peptides and, in particular, spatial–spectral mapping of not only changes in peptide conformation and association but also structural ordering or disordering of the membrane lipids upon binding or association. These studies could address questions around lipid domain targeting, or how membrane association can induce conformational changes (26). Indeed, the technology for superresolved IR spectroscopy continues to expand with the recent development of optically based PT IR, which is showing promise for obtaining IR spectra of subcellular structures in live cells (27, 28). These are, indeed, exciting times for the field as we look to the next chapter in this story.
